# Recruitment of MAIT Cells to the Intervillous Space of the Placenta by Placenta-Derived Chemokines

**DOI:** 10.3389/fimmu.2019.01300

**Published:** 2019-06-06

**Authors:** Martin Solders, Laia Gorchs, Eleonor Tiblad, Sebastian Gidlöf, Edwin Leeansyah, Joana Dias, Johan K. Sandberg, Isabelle Magalhaes, Anna-Carin Lundell, Helen Kaipe

**Affiliations:** ^1^Clinical Immunology and Transfusion Medicine, Karolinska University Hospital, Stockholm, Sweden; ^2^Department of Laboratory Medicine, Karolinska Institutet, Stockholm, Sweden; ^3^Center for Fetal Medicine, Karolinska University Hospital and Department of CLINTEC, Karolinska Insitutet, Stockholm, Sweden; ^4^Department of Women's and Children's Health, Karolinska Institutet, Stockholm, Sweden; ^5^Center for Infectious Medicine, Department of Medicine, Huddinge, Karolinska Institutet, Stockholm, Sweden; ^6^Program in Emerging Infectious Diseases, Duke-National University of Singapore Medical School, Singapore, Singapore; ^7^Department of Oncology/Pathology, Karolinska Institutet, Stockholm, Sweden; ^8^Department of Rheumatology and Inflammation Research, Sahlgrenska Academy, University of Gothenburg, Gothenburg, Sweden

**Keywords:** MAIT cells, placenta, chemokines, reproductive immunology, intervillous space, T cells, MIF

## Abstract

The intervillous space of the placenta is a part of the fetal-maternal interface, where maternal blood enters to provide nutrients and gas exchange. Little is known about the maternal immune cells at this site, which are in direct contact with fetal tissues. We have characterized the T cell composition and chemokine profile in paired intervillous and peripheral blood samples from healthy mothers giving birth following term pregnancies. Mucosal-associated invariant T (MAIT) cells and effector memory (EM) T cells were enriched in the intervillous blood compared to peripheral blood, suggesting that MAIT cells and other EM T cells home to the placenta during pregnancy. Furthermore, pregnant women had lower proportions of peripheral blood MAIT cells compared to non-pregnant women. The levels of several chemokines were significantly higher in intervillous compared to peripheral blood, including macrophage migration inhibitory factor (MIF), CXCL10, and CCL25, whereas CCL21, CCL27 and CXCL12 were lower. Migration assays showed that MAIT cells and EM T cells migrated toward conditioned medium from placental explants. A multivariate factor analysis indicated that high levels of MIF and CCL25 were associated with high proportions of MAIT cells in intervillous blood. Blocking of MIF or a combination of MIF, CCL25, and CCL20 in migration assays inhibited MAIT cell migration toward placenta conditioned medium. Finally, MAIT cells showed migratory capacities toward recombinant MIF. Together, these findings indicate that term placental tissues attract MAIT cells, and that this effect is at least partly mediated by MIF.

## Introduction

During pregnancy, one of the main functions of the placenta is to provide the growing fetus with oxygen and nutrients from the maternal blood circulation. The pregnant woman's arterial blood fills the intervillous space of the placenta, where it comes in direct contact with the fetal villi protruding from the fetal part of the placenta. Fetal blood vessels run inside the villi, and gas and nutrients are exchanged over a thin membrane of fetal cytotrophoblasts and syncytiotrophoblasts (1). Another site for fetal-maternal interactions is the decidua, a maternal membrane reformed from the endometrium during pregnancy. The decidua is readily infiltrated by maternal immune cells that can interact with invasive fetal extravillous trophoblasts [reviewed in (2)].

The circulation of maternal blood in the intervillous space from a physiological perspective has been described (3). The intervillous blood (IVB) is exchanged 2–3 times per minute (1), suggesting that the cell composition in IVB may reflect that of peripheral blood (PB). However, our recent findings showed that mucosal-associated invariant T (MAIT) cells and effector memory (EM) T cells are enriched in the IVB of term placentas (4), indicating that certain immune cell subsets are recruited to or retained in the intervillous space. The potential factors involved in the migration of maternal immune cells to the placenta is still unexplored.

MAIT cells are an invariant type of T cell that respond to microbial derived metabolites from riboflavin synthesis presented by the non-polymorphic MHC class I related molecule (MR1) (5). The MAIT cell T cell receptor invariably uses the Vα7.2-segment coupled with either Jα33, 12 or 20, paired with a limited set of β chains (6–8). Besides the MR1- and T cell receptor-dependent activation, MAIT cells can be partially activated or co-stimulated by cytokines, including IL-7, IL-12, IL-15, and IL-18 (8–11). Thus, MAIT cells can respond in a T cell receptor-independent manner, broadening their relevance to viral infections and autoimmune disorders (12–15). Upon activation, MAIT cells secrete IFN-γ and TNF-α, (16, 17) and MAIT cells from the liver and the female genital tract also secrete measurable levels of IL-17 and IL-22 (18, 19). Activated MAIT cells upregulate the expression of the cytotoxic effector molecules granzyme B and perforin, and can kill infected target cells in an MR1-restricted manner (20). The importance of MAIT cells in pregnancy is unknown.

Placental tissue and fetal trophoblasts produce a wide array of chemokines in early pregnancy (21), and chemokines and their receptors play an instrumental role in trophoblast invasiveness, angiogenesis, and recruitment of immune cells to the decidua [reviewed in (22)]. Macrophage migration inhibitory factor (MIF) is a chemokine-like cytokine highly expressed in placental tissues (23, 24). MIF expression has been shown in syncytiotrophoblasts, cytotrophoblasts and extravillous trophoblasts (23, 25), but its function in pregnancy is unclear.

Since particular immune cell subsets are enriched in IVB compared to PB (4), we hypothesized that the placenta produces chemotactic mediators that are involved in attracting certain maternal immune cells to the intervillous space. Therefore, the main aim of this study was to examine if placental-derived factors are involved in recruiting MAIT cells to the placenta.

## Materials and Methods

### Sample Collection

Healthy individuals (*n* = 36, median age 34, range 21–42) donated their placentas after informed consent, subsequent to planned cesarean sections following uncomplicated term pregnancies (median gestational week 39, range 38–42). The regional review board of ethics in research at Karolinska Institutet approved the donation of peripheral blood and placentas (entry numbers 2009/418-31/4, 2010/2061-32, and 2015/1848-31/2). Female blood donors were used as a control for non-pregnant women (*n* = 27, median age 46, range 22–71).

Data on MAIT cell frequencies from pregnant women has been published previously for 21 out of 36 donors (4). Parts of the T- and MAIT cell phenotype data were published previously for 11 out of 36 donors (4).

### Cell Isolation

Peripheral blood (PB) samples were collected a few hours before the caesarian sections. The description of isolation of IVB and decidua parietalis has been published previously (4). Briefly, placentas were placed in a sterile container in the operation room, and then taken directly to the lab. After removal of the fetal membranes and clamping of the umbilical cord, the placentas were placed with the maternal side facing up. Visible blood clots were removed, and the surface was washed with phosphate buffered saline (PBS) to remove seeping blood. The placenta was turned around and lifted so that the maternal side faced downwards and IVB was collected on a sterile petri dish and immediately transferred to heparin tubes. The samples of PB and IVB were centrifuged at 600 g for 8 min after which plasma samples were collected and stored at −80°C until use. The blood was diluted 1:2 with PBS, and mononuclear cells were isolated by density gradient centrifugation (Lymphoprep, Axis-Shield, Oslo, Norway). The fetal membranes were placed with the decidua facing up. After extensive washing and removal of visible blood clots, the decidua parietalis was mechanically scraped off from the chorion, pooled and washed with PBS by repeated short centrifugations. Cells were released from the tissue by using a GentleMACS Dissociator (Miltenyi Biotec, Bergisch Gladbach, Germany). After filtering and washing, mononuclear cells were isolated by density gradient centrifugation (Lymphoprep, Axis-Shield, Dundee, Scotland).

Cells were either stained directly for flow cytometry, or re-suspended in RPMI (HyClone, GE Health Sciences, South Logan, UT) medium supplemented with 10% fetal calf serum, 100 U/ml penicillin, 100 μg/ml streptomycin (complete medium) containing 10% DMSO and frozen in aliquots in liquid nitrogen. In our previous publication, we found the isolated maternal cell product to have a median fetal DNA contamination of 12.1% (range 5.2–19.4, *n* = 6) (4).

### Flow Cytometry

IVB and PB samples were analyzed in pairs, either fresh or thawed after freezing. Frozen cells were thawed in complete medium, washed in PBS and counted, and fresh cells were used directly after isolation. The cells were plated in a 96-well plate, ≤ 1 × 10^6^ cells/well. Staining was carried out in 50 μL of CliniMACS PBS/EDTA buffer (Miltenyi Biotech, Bergish Gladbach, Germany) supplemented with 0.1% bovine serum albumin (FACS-buffer) to which antibodies were added, and incubated for 30 min at 4°C. After washing, the cells were stained with 7AAD, which was used to distinguish live from dead cells. The MR1 tetramers were produced by the NIH Tetramer Core Facility as permitted to be distributed by the University of Melbourne, and the MR1 tetramer technology was developed jointly by Dr. James McCluskey, Dr. Jamie Rossjohn, and Dr. David Fairlie. The flow cytometry antibodies used in this study are listed in Supplementary Table S1. Data were collected using a BD FACSCanto flow cytometer and analyzed with FlowJo software (Tree Star, Ashland, OR, USA). Data on conventional T cells were analyzed after excluding CD161^+^ and Vα7.2^+^ T cells.

### Placenta Conditioned Medium

Placental explant conditioned medium were isolated using a slightly modified version of the method described for 1st trimester placentas by Svensson-Arvelund et al. (21). Following extraction of IVB, the placenta was placed with the maternal side facing up. Using scissors, the decidua basalis was removed, and from the underlying tissue, biopsies of approximately 1 cm^3^ were cut. Between 5 and 10 biopsies were taken from different places of the placenta. The tissue biopsies were then pooled and dissected into as small pieces as possible using forceps and a scalpel. Macroscopically identifiable blood vessels were removed. The dissected tissue was then washed extensively with PBS by repeated cycles of short centrifugations until the PBS was no longer colored by the tissue, usually 10–20 times. The tissue was then placed in 24- (50–100 mg of tissue) or 6-well plates (250–500 mg of tissue), and 10 μL of complete medium/mg of tissue was added. Multiple plates were set up at the same time, and incubated at 37°C for 48 and 72 h. At the end of incubation, the content of multiple wells was harvested and pooled, and following centrifugation (600 g, 8 min), supernatants were collected and frozen in aliquots at −80°C.

### Multiplex Chemokine Assay

Paired frozen plasma samples from IVB and PB (*n* = 25) and placental explant supernatants (*n* = 5) were analyzed for cytokine and chemokine concentrations using a Magpix with Luminex Xponent software (Luminexcorp, Austin, TX, USA) and the Bio-Plex Pro Human Chemokine 40-Plex kit (BIO-RAD, Hercules, CA, USA) according to the manufacturer's instructions. The detection level of each analyte is shown in Supplementary Table S2.

### Migration Assay

T cells were purified from PBMCs from healthy donors by negative selection (Pan T Cell Isolation Kit, Miltenyi Biotec, Bergisch Gladbach, Germany) according to the manufacturer's instructions. Placenta conditioned medium from 48 or 72 h cultures was thawed, diluted 50% in complete medium, and 600 μL was added to a 24-well plate. Complete medium only was used as a negative control. Blocking reagents against CCL20 (clone 67310, 1 μg/ml, R&D Systems, Minneapolis, MN, USA), CCL25 (clone 52513, 3 μg/ml, R&D) and/or MIF (ISO-1, 100 μM, Tocris Bioscience, Bristol, UK) or the corresponding isotype/diluent was then added to the wells. ISO-1 was diluted in PBS with 0.1% DMSO. For the MIF migration assay, RPMI medium without serum containing 100 or 250 ng/ml recombinant MIF (R&D Systems) were added to the lower well. The medium control well contained only RPMI without serum. Ten of the experiments were performed using purified T cells (isolated as described above). Six samples were performed by using purified T cells that were also depleted of CD4^+^ T cells, using CD4^+^ T cell isolation beads (Miltenyi), to enrich CD8^+^ T cells. Transwell inserts (5 μm pore size, Costar, Cambridge, MA, USA) were placed into each well, and 1 × 10^6^ T cells resuspended in 100 μL RPMI without serum were then added on top of the transwell insert. Plates were incubated at 37°C for 3 h. The transwell inserts were removed, and the content of each well was harvested, transferred to a 15 ml tube, centrifugated (600 g, 8 min), re-suspended in FACS-buffer, and stained for flow cytometry. Careful measures were taken to treat all paired samples equally; they were re-suspended in the same volume of FACS-buffer, washed in parallel, and run for the same time in the flow cytometer. Results are presented as cell counts or proportions within the CD3^+^ or CD8^+^ T cell populations as indicated in the figures.

### Statistical Analysis

**Figure 1:**
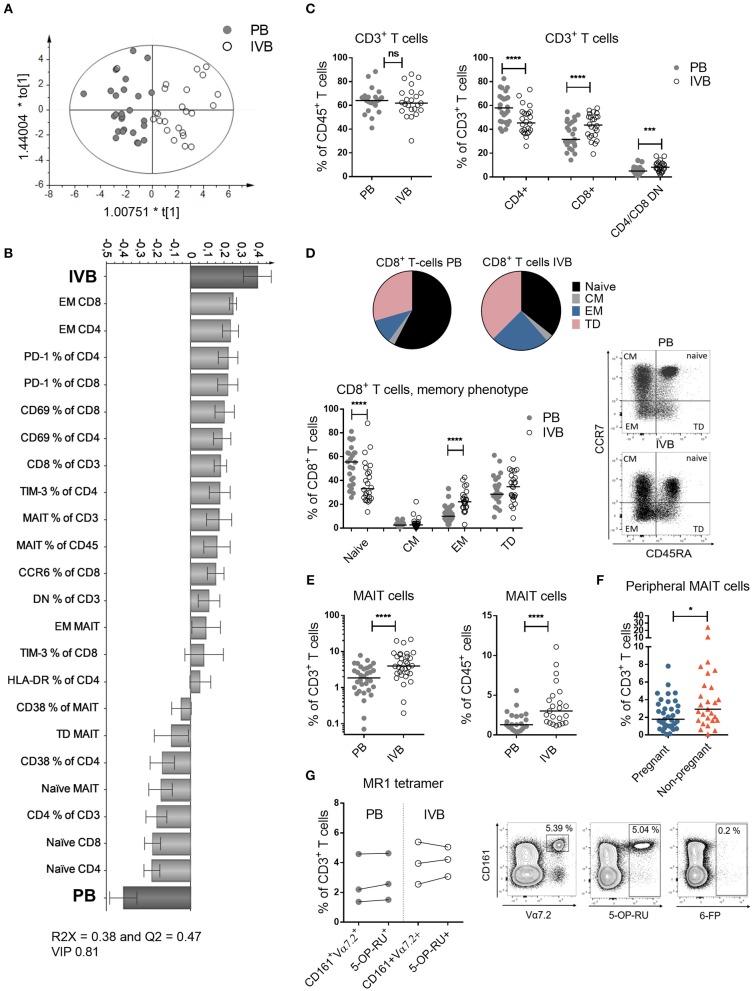
T cell subsets and phenotypes are different in intervillous compared to peripheral blood. **(A)** OPLS-DA observation plot displaying a maximized separation between peripheral (PB) and intervillous blood (IVB) based on T cell phenotypes along the predictive component t[1] and the orthogonal component to[1] accounts for within class variability (PB, *n* = 25, IVB, *n* = 24). **(B)** OPLS-DA loading plot based on the same data as in **(A)**, following a variable influence of projection (VIP) of 0.81 (Supplementary Figure S1), showing associations between IVB or PB and phenotypic markers on T cells. **(C)** Proportions of CD3^+^ cells within CD45^+^ cells (left), and CD4^+^, CD8^+^ and CD4^−^/CD8^−^ cells within CD3^+^ cells compared between PB and IVB (right) (*n* = 24). **(D)** Median proportions of naïve, central memory (CM), effector memory (EM) and terminally differentiated (TD) cells within CD8^+^ T cells, based on the co-expression of CCR7 and CD45RA depicted in a pie chart (top) and as individual values (bottom) (*n* = 24). Representative dot plots are shown to the right. **(E)** Percentage of MAIT cells out of total CD3^+^ cells (left, *n* = 24) and CD45^+^ cells (right, *n* = 23) compared between PB and IVB. **(F)** Percentage of MAIT cells out of total CD3^+^ cells compared between peripheral blood from pregnant (*n* = 35) and non-pregnant women (*n* = 27). **(G)** Comparison of MAIT cell characterization methods between CD161^+^Vα7.2^+^ and 5-OP-RU tetramers in PB and IVB (left, *n* = 3), and representative plots of the stainings, including the negative control tetramer loaded with 6-FP (right). Line in graphs represents the median. Comparisons between paired samples were performed by Wilcoxon test **(C–E** and **G)** and unpaired samples by Mann-Whitney test **(F)**. ns = not significant, ^*^p < 0.05, ^***^p < 0.001, ^****^p < 0.0001.

We used multivariate orthogonal projection to latent structures (OPLS) analyses as a tool to screen for differences between PB and IVB and associations between specific immune cell subsets and chemokine levels. Variables that showed the strongest association to classes or variables were further evaluated by univariate analysis. Multivariate orthogonal projection to latent structures by means of partial least squares discriminant analysis (OPLS-DA) was used to obtain a maximum separation of X-variables (T cell phenotypes or chemokine levels) based on class information (peripheral and intervillous blood, i.e., PB and IVB) (SIMCA software, Sartorius Stedim Biotech, Umeå, Sweden). The two-class (PB and IVB) discrimination OPLS-DA model presented in Figure 1A is a default score scatter observation plot with one predictive component t[1] and one orthogonal component to [1]. The scatter plot of t[1] vs. to[1] is a window in the X space in which the separation of the two classes of observations occurs in the horizontal t[1] direction and the within class variability is expressed in the vertical to[1] direction. Linear OPLS analysis was implemented to investigate associations between a selected Y-variable (proportion of MAIT cells) and a set of X-variables (chemokine levels in IVB). The contribution of each X-variable, variable influence of projection (VIP) values, to the OPLS models were calculated. For the analysis of T cell variabilities, X-variables with a VIP value below 0.81 were excluded and a new model was generated based on remaining variables. The scale presented on the x and y-axis in the OPLS-DA plot (Figure 1A) and on the y-axis of the OPLS plots (Figures 1B, **4A**) is a dimensionless scale in which the loading vector is normalized to unit length. The validation of OPLS analyses is based on the goodness of fit, or R2, which tells how well the data set can be mathematically reproduced. The goodness of predictivity, or Q2, tells how well the model can predict future data of a test set. Cross validation is performed by SIMCA software to estimate Q2. Briefly, cross validation is performed by dividing the data set into a number of groups and then developing a number of parallel models from the reduced data with one of the groups deleted. After developing a model, the deleted data are used as a test set, and differences between actual and predicted values are calculated for the test set, eventually leading forward to the Q2-value. Logarithmic values were used in the multivariate analysis. The factors contributing most to the separation were further analyzed using the two-tailed Wilcoxon matched-pairs signed rank test. To investigate correlations between two factors, the Spearman's rank correlation test was used. To detect differences across two groups of unpaired samples, the two-tailed Mann-Whitney test was used (GraphPad Software, La Jolla, CA). A *P* of < 0.05 was considered significant.

## Results

### T Cell Subsets and Phenotypes Are Different in Intervillous Compared to Peripheral Blood

By using multivariate discriminant analysis, we first examined the T cell profiles in PB and IVB based on 53 parameters, which are all displayed in Supplementary Figure S1. As depicted in the observation plot in Figure 1A, a clear separation was found between PB and IVB based on the T cell variables assessed. The *X*-variables that showed the strongest associations with PB or IVB are shown in the loading plot in Figure 1B, a model based on *X*-variables with VIP values ≥ 0.81. A VIP column plot for all analyzed parameters is shown in Supplementary Figure S1. Further investigating the two groups using univariate statistical analysis, we found several significant differences in the T cell compartment between PB and IVB. The proportions of CD3^+^ T cells within CD45^+^ cells were similar in PB and IVB, but IVB had higher proportions of CD8^+^ and CD4 and CD8 double negative (DN) T cells compared to PB (Figure 1C). The proportion of EM CD8^+^ T cells was a median 2.1-fold higher in IVB compared to PB, with a concomitant lower proportion of naïve T cells (Figure 1D). A similar pattern was observed among CD4^+^ T cells, with a 1.7-fold higher proportion of EM cells in IVB compared to PB (data not shown). CD8^+^ T cells from IVB were more activated compared to PB CD8^+^ T cells, as shown by an increased expression of CD69 and HLA-DR (Supplementary Figure S2A). IVB CD8^+^ T cells also expressed the co-inhibitory markers PD-1 and TIM-3 to a larger degree compared to PB CD8^+^ T cells (Supplementary Figure S2A). Taken together, this shows that maternal T cells in the intervillous space to a larger degree are activated and of an EM phenotype compared to their peripheral counterparts. Representative flow cytometry plots are shown in Supplementary Figure S3.

### Enrichment of MAIT Cells in Intervillous Blood

As previously shown (4), but now with a larger number of donors, MAIT cells were enriched in IVB both within CD3^+^ T cells and within CD45^+^ leukocytes, with a median 2.1-fold and 2.3-fold higher proportion, respectively, in IVB compared to PB (Figure 1E). This suggests that MAIT cells accumulate in the intervillous space of the placenta during pregnancy. The proportion of MAIT cells in PB from the cohort of pregnant women was compared to that of healthy non-pregnant women. Despite the lower median age of the pregnant compared to the non-pregnant women (34 vs. 46 years, respectively), the non-pregnant women had significantly higher levels of MAIT cells in PB compared to pregnant women (Figure 1F). Together, this suggests that a proportion of MAIT cells may leave the circulation and enter the placenta during late healthy pregnancy. The proportion of CD4^+^, CD8^+^ or CD4/CD8 DN conventional T cells did not significantly differ between pregnant and non-pregnant women (Supplementary Figure S4). Data on the proportion of conventional EM T cells was not available in this cohort of non-pregnant women.

Throughout this study, MAIT cells were defined by the expression of CD161 and Vα7.2. To confirm that these cells were MR1-restricted, we stained PB and IVB cells from three donors with the MR1 5-OP-RU tetramers. It showed that similar proportions of MAIT cells were found when using the MR1 tetramer or the CD161 and Vα7.2 gating strategy both in PB and IVB (Figure 1G). Out of the CD161^+^Vα7.2^+^ cells, a median of 93.6% and 93.9% stained positive for the MR1 tetramer in PB and IVB, respectively (Supplementary Figure S2B). The negative control tetramer MR1 6-FP showed little or no staining (Figure 1G).

The majority of MAIT cells in both IVB and PB in the pregnant women were CD8^+^, but intervillous MAIT cells were to a larger degree CD4 and CD8 DN and to a lower extent CD4^+^ compared to peripheral MAIT cells (Supplementary Figure S2C). This pattern was similar when the cells were gated on MR1 tetramer^+^ cells in three donors (Supplementary Figure S2D). As observed for the conventional T cells, intervillous MAIT cells had more uniform EM characteristics as compared to peripheral MAIT cells (Supplementary Figure S2E). In contrast to conventional CD8^+^ T cells, IVB MAIT cells had a less activated phenotype compared to PB MAIT cells with lower expression of CD25 and PD-1, but similar expression of CD69 and HLA-DR (Supplementary Figure S2F). Together, intervillous and peripheral MAIT cells display different phenotypic patterns, and conventional T cells and MAIT cells show relative differences between IVB and PB in the expression patterns of both early and late activation markers.

### Placental Tissues Produce High Levels of Several Chemokines Which Attracts MAIT Cells and CD8^+^ Effector Memory T Cells

**Figure 2:**
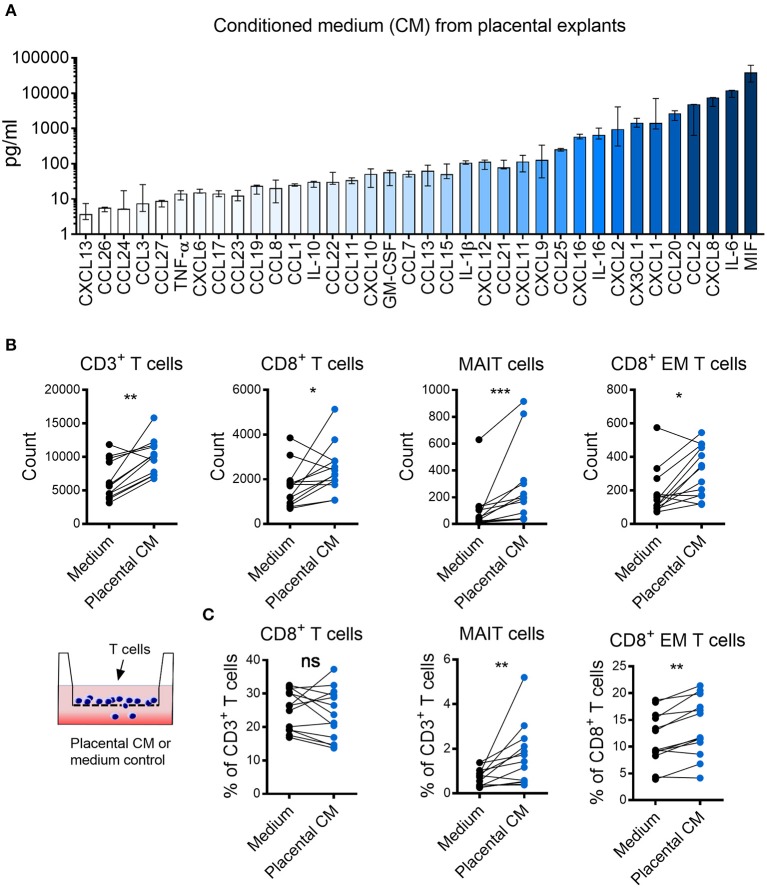
Placental tissues produce high levels of several chemokines that attract MAIT cells and CD8^+^ effector memory T cells. **(A)** Chemokine levels in conditioned medium (CM) from placental tissue explants. Bars depict median values, error bars represent the range (*n* = 5). **(B,C)** Comparisons between migration of purified CD3^+^ T cells toward placental (CM) and medium control. **(B)** Number of migrated CD3^+^ cells (left), CD8^+^ cells (middle left), MAIT cells (middle right), and CD8^+^ effector memory (EM) T cells (right). **(C)** Percentage of CD8^+^ cells (left) and MAIT cells (middle) within the CD3^+^ population and percentage of CD8^+^ EM cells within the CD3^+^CD8^+^ population (right). Paired experiments are connected by a line. Comparisons between paired samples were made using the nonparametric Wilcoxon test. ns, not significant, ^*^p < 0.05, ^**^p < 0.01, ^**^p < 0.001.

We hypothesized that the observed enrichment of certain T cell populations in the intervillous space of the placenta was due to chemotactic factors produced by the placental tissue. Therefore, we examined the chemokine secretion pattern from placental explants. Several small pieces of placental villous tissue were collected and pooled from the same placenta to provide a representative sample. The tissue was cultured for 48 h, and the supernatant was harvested and analyzed by a 40-plex luminex assay. As shown in Figure 2A, placental explants secreted high levels of several chemokines and cytokines, including MIF, IL-6, CXCL8, and CCL2. To investigate the potential chemoattractive capacity of placental tissue on MAIT cells and other T cells, an assay of immune cell migration toward placental tissue conditioned medium was set up. We found that the placental tissue conditioned medium promoted migration of CD3^+^ T cells, CD8^+^ T cells, MAIT cells, and CD8^+^ EM T cells, as compared to control medium (Figure 2B). The number of migrating CD4^+^ T cell, DN T cells, as well as CD8^+^, DN and CD4^+^ MAIT cells all increased in the presence of conditioned medium (Supplementary Figures S5A,B). The proportion of migrating CD8^+^, CD4^+^ and DN T cells out of CD3^+^ T cells was not altered when placenta conditioned medium and control medium was added (Figure 2C; Supplementary Figure S5A). On the other hand, the proportion of both MAIT cells and conventional CD8^+^ EM T cells were higher when placenta conditioned medium was added, compared to control medium (Figure 2C). The proportion of CD8^+^ and DN migrating MAIT cells was not significantly affected by the conditioned medium, but the percentage of CD4^+^ MAIT cells were lower in MAIT cells migrating toward the conditioned medium (Supplementary Figure S5B). No significant effect was observed for the proportion of CD4^+^ EM T cells (data not shown). Thus, this indicates that chemokines produced from fetal placental tissues attracts T cells in general, but have a stronger attracting capacity on MAIT cells and conventional CD8^+^ EM T cells compared to other T cell subsets.

### Chemokine Profile in Intervillous Blood Is Different From That of Peripheral Blood

**Figure 3:**
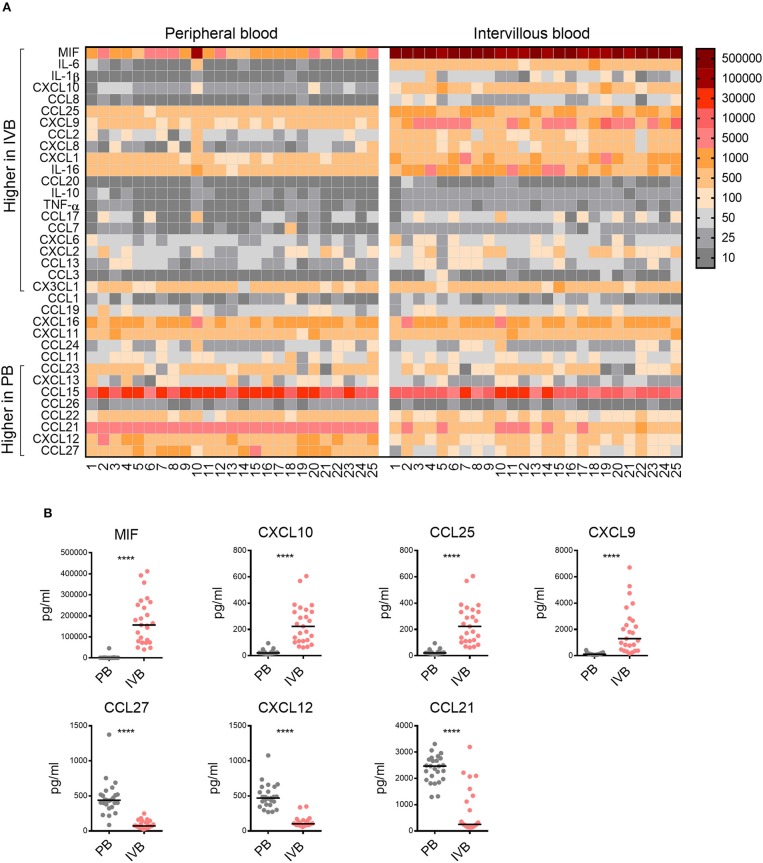
Chemokine profile in intervillous blood is distinct from that of peripheral blood. **(A)** Heatmap displaying the levels of chemokines and cytokines (pg/ml plasma) in all individual samples of peripheral (PB) and intervillous blood (IVB) (*n* = 25). The brackets indicate significant differences between PB and IVB. Numbers indicate individual donors. **(B)** The levels of MIF, CXCL10, CXCL9, and CCL25 were significantly higher in IVB compared to PB, whereas the levels of CCL27, CXCL12, and CCL21 where significantly lower. Line in graphs represents the median. Comparisons between paired samples were made using the nonparametric Wilcoxon test. ^****^p < 0.0001.

Following our findings regarding the chemoattractive properties of term placental tissue, we investigated the chemokine pattern in the blood adjacent to the fetal villi in the intervillous space. Plasma from paired PB and IVB samples were analyzed to examine the chemokine profile in these compartments. As shown in the OPLS-DA loading plot in Supplementary Figure S6, a large proportion of the investigated chemokines were associated to either PB or IVB. The levels of chemokines and cytokines in PB and IVB in all individual samples are displayed in a heatmap, sorted based on the results from the OPLS-DA (Figure 3A). Univariate analysis showed significantly higher levels of a majority of the analyzed soluble factors in IVB compared to PB, whereas other were higher in PB, as indicated in Figure 3A. MIF displayed the strongest association with IVB and showed a median 182-fold higher level in IVB compared to PB (Figure 3B). Other factors that were higher in IVB included CXCL10, CCL25 and CXCL9 (Figure 3B). In contrast, levels of CCL27, CXCL12, and CCL21 were significantly lower in IVB compared to PB. Many of the chemokines that were produced in high levels from the placental explants (Figure 2A), were higher in IVB than PB plasma (Figures 3A,B). A direct comparison between the levels of chemokines in placental conditioned medium and IVB plasma are shown side by side in Supplementary Figure S7A. The relative differences between levels of chemokines in IVB and PB plasma, as determined by the ratio of levels in IVB divided by levels in PB, are shown in Supplementary Figure S7B. Together, this suggests that the placental villous tissue was responsible for the production of at least some of the chemokines detected at elevated levels in IVB plasma.

### MIF Levels in Intervillous Blood Correlate With the Proportion of MAIT Cells in the Placenta

**Figure 4:**
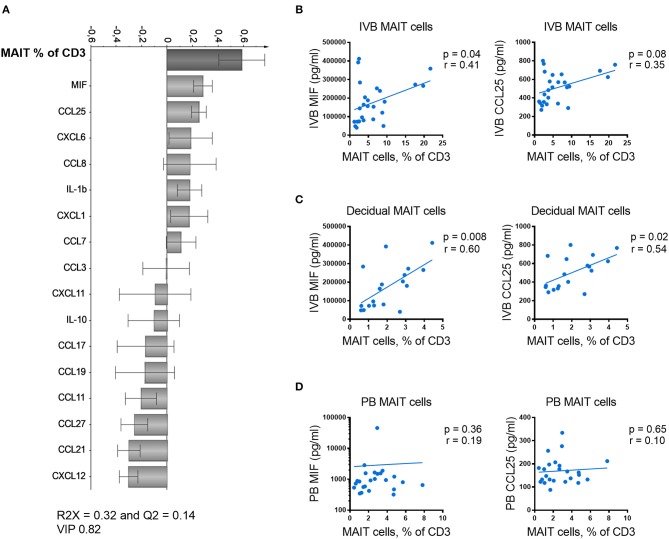
MIF levels in intervillous blood correlate with proportions of MAIT cells in the placenta. **(A)** Loading plot from linear OPLS analysis showing associations between the frequency of intervillous blood (IVB) MAIT cells and paired data on chemokine and cytokine levels in IVB. **(B)** Correlation between the frequency of IVB MAIT cells and the measured levels of MIF (left) and CCL25 (right) in paired samples from IVB (*n* = 25). **(C)** Correlation between the frequency of MAIT cells in decidua parietalis and the measured levels of MIF (left) and CCL25 (right) in paired IVB samples (*n* = 18). **(D)** Correlation between the frequency of peripheral blood (PB) MAIT cells and the measured levels of MIF (left) and CCL25 (right) in paired PB plasma samples (*n* = 24). Correlations between paired samples were made using the nonparametric Spearman correlation test.

The data on paired IVB plasma chemokine levels and IVB T cell composition allowed us to study associations between particular T cell subsets and levels of placenta-derived chemokines. In an attempt to identify factors related to MAIT cell homing to the intervillous space, the proportions of MAIT cells in IVB were analyzed for associations with levels of IVB chemokine levels using OPLS analysis. As shown in Figure 4A, higher proportions of MAIT cells were positively associated with higher levels of MIF and CCL25 (Figure 4A). In univariate analysis, the proportion of MAIT cells in IVB correlated directly to the levels of MIF in IVB, and there was a trend for correlation with CCL25 levels (Figure 4B). Paired data on proportions of MAIT cells from decidua parietalis and IVB chemokine levels was available from 18 of the donors, and showed an even stronger correlation with levels of both MIF and CCL25 in IVB (Figure 4C). These associations further support the notion that placenta-derived MIF and CCL25 are involved in attracting maternal MAIT cells to both the decidua and the intervillous space. In contrast, when analyzing the association between proportions of MAIT cells in PB with levels of MIF and CCL25 in the same samples, no correlation was found (Figure 4D). Thus, MIF and CCL25 did not appear to affect MAIT cell numbers in general, but rather seemed to be involved in directing MAIT cells from the periphery into the placental intervillous space and to the decidua. IVB plasma levels of CCL21 and CCL27 were inversely correlated to IVB MAIT cell proportions (Supplementary Figure S8A).

Conventional CD8^+^ EM T cells in IVB showed no correlation with IVB MIF or CCL25 levels (Supplementary Figure S8B). However, the proportions of conventional CD8^+^ EM T cells instead were significantly correlated to the levels of the CXCR3-ligands CXCL9, CXCL10, and CXCL11 (Supplementary Figure S8C). This suggests that MAIT cells and conventional CD8^+^ EM T cells are recruited to the intervillous space by different chemokine gradients.

### Placenta-Derived Chemokines and MIF Promote MAIT Cell Migration

**Figure 5:**
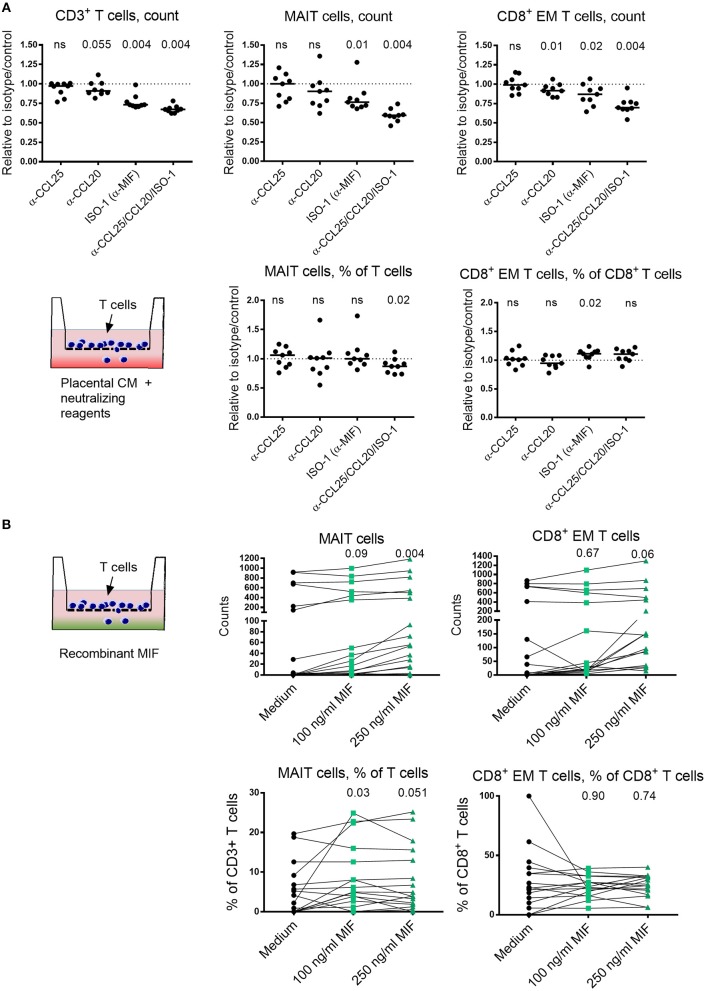
Placenta-derived chemokines and MIF promote MAIT cell migration. **(A)** The effect of blocking CCL25, CCL20 and MIF, alone or all together, on the number of migrating CD3^+^ T cells (top left), MAIT cells (top middle) or CD8^+^ effector memory (EM) T cells (top right), toward placental conditioned medium (CM). The proportion of migrating MAIT cells within CD3^+^ T cells (bottom left) and CD8^+^ EM T cells among CD8^+^ T cells (bottom right) toward CM. Results are expressed as blocking relative to isotype or diluent control. **(B)** The chemoattractive effect of MIF on the migration of number of MAIT cells (top left) and CD8^+^ EM T cells (top right), using increasing concentrations of MIF. Proportion of migrated MAIT cells within CD3^+^ T cells (bottom left) and CD8^+^ EM T cells among CD8^+^ T cells (bottom right) in the absence and presence of recombinant MIF. *P* values refer to comparisons between the medium control and the indicated levels of MIF. **(B)** The data on the bottom y-axis for MAIT and CD8^+^ EM T cell counts were generated when adding purified T cells and the top y-axis when using CD4-depleted T cells. Line in graphs represents the median. Comparisons between paired samples were made using the nonparametric Wilcoxon test. ns, not significant.

To further investigate the relationship between IVB MAIT cells and chemoattractive factors, we next blocked CCL25 and MIF, identified as putatively important for MAIT cell homing to the placenta, in a migration assay. Neutralizing antibodies to CCL25 or the MIF-inhibiting chemical ISO-1 were used to block these factors in the placental tissue conditioned medium. Since the majority of the MAIT cells are highly positive for CCR6, the receptor for CCL20, we also included neutralizing antibodies to this chemokine. For total CD3^+^ T cells, we found that the number of migrating T cells were reduced when ISO-1 or the combination of all three factors were added to the conditioned medium (Figure 5A). A similar pattern was observed for the number of migrating MAIT cells. Among the three conditions, only ISO-1 significantly decreased the migration of MAIT cells with a median of 24 % relative to the diluent control (Figure 5A). When all three factors were blocked, the number of migrating MAIT cells decreased with a median of 41% relative to isotype and diluent control. When analyzing the proportion of MAIT cells within migrating CD3^+^ T cells, we found that the MAIT cell proportions were significantly reduced when all three factors were blocked, whereas there was no significant effect on MAIT cell proportion when only anti-CCL25, anti-CCL20, or ISO-1 were added on their own (Figure 5A).

For CD8^+^ EM T cells, we found that the number of cells that migrated was significantly blocked by ISO-1, but only with a median of 13% relative to the control (Figure 5A). Neutralization of CCL20 also led to a statistically significant, but small decrease in migration. Blocking of all three factors decreased the number of migrating CD8^+^ EM T cell with a median of 30 % relative to control. However, when the proportion of CD8^+^ EM T cells among total migrating CD8^+^ T cells was analyzed, we found no significant reduction in migration by blockade of any of the factor, either alone or in combination. Instead, an increase in CD8^+^ EM T cell proportion was observed when ISO-1 was added (Figure 5A). Thus, MIF appear to attract T cells in general, since the number of total migrating T cells were reduced when ISO-1 was added. However, the proportion of CD8^+^ EM T cells among migrating CD3^+^ T cells was increased when MIF was blocked, suggesting that MIF has little specific effect in promoting migration of CD8^+^ EM T cells as compared to other T cell subsets. On the other hand, the proportion of MAIT cells among migrating T cells was reduced when blockade of MIF in combination with CCL25 and CCL20 was performed. Raw data on cell counts after addition of isotype controls, diluent controls, and neutralizing agents are shown in Supplementary Figure S9.

Since MIF appeared to be an important factor for MAIT cell attraction, we next examined if recombinant MIF could promote migration of T cells and MAIT cells. MIF significantly increased the number of migrating MAIT cells at a concentration of 250 ng/ml (Figure 5B). However, MIF did not promote migration of conventional CD8^+^ EM T cells as determined by the number of migrating cells, although there was a trend at the higher concentration of 250 ng/ml (Figure 5B). When analyzing the proportion of MAIT cells and CD8^+^ EM T cells among migrating T cells in the parent gate, we found that only MAIT cells were significantly increased when MIF was added and no effect was found for CD8^+^ EM T cells (Figure 5B). Sufficient counts were recorded in seven of the donors to allow sub-gating. For these, we found that the proportion of migrating CD8^+^ MAIT cells were reduced when MIF was added, and there was a trend for an increased proportion of migrating DN MAIT cells (Supplementary Figure S10). Thus, these findings support the notion that MIF has a T cell attracting capacity and that this effect is stronger on MAIT cells than on conventional CD8^+^ EM T cells.

### MAIT Cells Express Higher Levels of CXCR4 Compared to CD8^+^ Effector Memory T Cells

**Figure 6:**
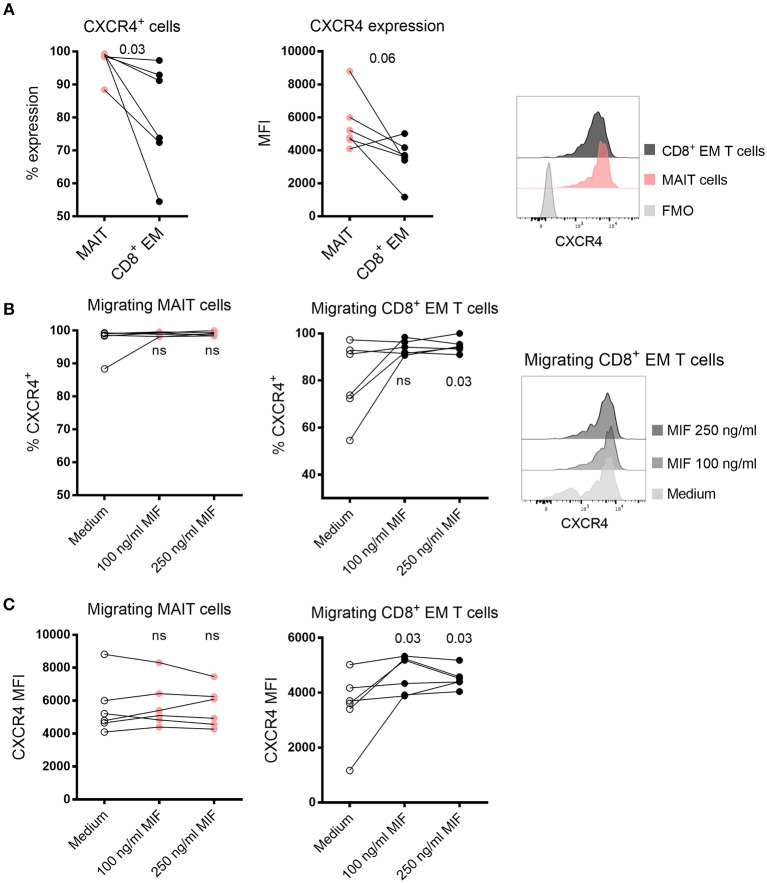
Expression of CXCR4 in migrating MAIT cells and CD8^+^ EM T cells. **(A)** The proportion (left) and median fluorescence intensity (MFI) (right) of CXCR4 expression in MAIT cells and CD8^+^ EM T cells in medium control. **(B)** The proportion of migrating CXCR4^+^ MAIT cells (left) and CD8^+^ EM T cells (right) after addition of recombinant MIF at 100 ng/ml or 250 ng/ml in migration assays. **(C)** The MFI of CXCR4 expression in migrating MAIT cells (left) and CD8^+^ EM T cells (right) in the presence of recombinant MIF. *P* values in B and C refer to comparisons between the medium control and the indicated levels of MIF. Representative histograms of CXCR4 expression are shown on **(A)** MAIT cells and CD8^+^ EM T cells and **(B)** and migrating CD8^+^ EM T cells toward medium control or MIF. Comparisons between paired samples were made using the nonparametric Wilcoxon test. ns, not significant.

MIF has previously been reported to bind to CXCR4 (26), and we next compared the level of CXCR4 expression on MAIT cells and CD8^+^ EM T cells in medium control wells. Although a large proportion of both MAIT cells and CD8^+^ EM T cells expressed CXCR4, the proportion of MAIT cell expressing CXCR4 was significantly higher (Figure 6A). The median intensity expression (MFI) of CXCR4 was also higher in MAIT cells in five out of six samples (Figure 6A). When MIF was added to the migration assay, we found that the proportion of CXCR4^+^ CD8^+^ EM T cells increased compared to medium control and also that the intensity of expression of CXCR4 was higher (Figures 6B,C). We found no enrichment of CXCR4-expressing MAIT cells when MIF was added (Figures 6B,C), but this could be explained by that the median expression of CXCR4 on MAIT cells was as high as 99 % in the control condition, a finding also supported by Dias et al. (27). In summary, the higher expression of CXCR4 on MAIT cells may increase the capacity to more efficiently migrate toward MIF compared to CD8^+^ EM T cells, and our data support that CXCR4 is involved in the migration of T cells toward MIF.

## Discussion

The decidua and the intervillous space are both sites for fetal-maternal immune interactions where maternal immune cells are in contact with fetal extravillous trophoblasts and syncytiotrophoblasts, respectively. Several studies have examined the composition of maternal immune cells in decidual tissues, in both healthy early and term pregnancies (28–30), as well as in miscarriage and pre-eclampsia (31, 32). In contrast, surprisingly little is known about immune cells in the IVB. In the present study, we show that the T cell compartment in IVB markedly differs from that in PB at term pregnancy, and that placenta-derived factors may play a role in recruiting or retaining certain T cell subsets in the intervillous space and decidua.

The multivariate discriminant analysis showed that high proportions of conventional EM T cells and MAIT cells, and activated conventional T cells were strongly associated to IVB. Non-pregnant women had a higher proportion of MAIT cells in the circulation compared to our cohort of pregnant women, but it should be noted that the groups were not age-matched and the sample collection from the non-pregnant women were not adjusted to their menstrual cycles, which could affect the results. The non-pregnant women had a higher median age, and since it has been shown that MAIT cell proportions decrease with age (33), the age difference may mask an even bigger decline in circulating MAIT cell levels in pregnancy. Our findings are thus consistent with a possible homing of peripheral MAIT cells to the placenta during pregnancy. We have previously shown that IVB MAIT cells do not express the proliferation marker Ki67 (4), which further indicate that MAIT cells are recruited or retained rather than proliferating at the site. To further study whether the increase in IVB MAIT cell frequency is due to homing from PB, it would be interesting to follow PB MAIT cell frequencies longitudinally, including before, during, and after pregnancy.

The chemokine pattern in IVB was clearly different from that of PB, and we also found that placental villous explants produced high levels of several inflammatory chemokines. Svensson-Arvelund et. al. (21) and others [reviewed in (22)], have previously shown that first trimester placental tissues and trophoblasts produce several chemokines, but very little is known about how placenta-derived chemokines promote immune cell migration. Nancy et al. showed that murine decidual stromal cells undergo epigenetic modifications that decrease the production of the CXCR3-ligands CXCL9, CXCL10, and CXCL11, which prevented effector T cells from entering the decidua in mouse models (34). The number of T cells in human first trimester decidua is low, (28, 29) but the composition of immune cells changes during pregnancy and T cells become as numerous in decidua as in peripheral blood at term (29, 30). It is therefore reasonable to believe that the expression of chemokines changes as pregnancy proceeds. Our data from term placental villous explant medium and intervillous plasma indicate that the term placenta produces a wide array of chemokines and cytokines, which can be involved in recruiting inflammatory T cells to the decidua and intervillous space at term.

By using a multivariate factor analysis, we found MIF to be the factor most strongly associated with high MAIT cell proportions in IVB. Considering the large inter-individual proportions of MAIT cells and the elevated levels of a large number of chemokines in IVB, we were surprised to find correlations between MAIT cell proportions and particular chemokine levels. However, the migration assays further supported that MIF was important for MAIT cell migration. Interestingly, decidual MAIT cell proportions from paired samples were strongly associated to MIF levels in the IVB, suggesting that MIF may also be involved in directing MAIT cell into adjacent tissues, and not only to retain them in a blood-filled space. However, it is likely that a combination of several chemokines ultimately regulates retention and migration of cells to a particular tissue, and that other chemokines may be of greater importance in settings other than pregnancy.

It was further evident that MAIT cells and conventional CD8^+^ EM T cells were associated with different chemokine patterns. Whereas proportions of MAIT cells were associated to high MIF and CCL25 levels, conventional CD8^+^ EM T cells correlated to the CXCR3-ligands CXCL9, CXLC10 and CXCL11. These findings support the results by Nancy et al. showing that the CXCR3-binding chemokines are important for attracting effector T cells to the placenta, but may also contradict the findings that these chemokines are silenced at the fetal maternal interface during pregnancy (34). Powell et al. observed an increased frequency of CXCR3^+^ CD4^+^ T cells in human 3rd trimester decidua compared to PB following uncomplicated pregnancies (35). Together, this points to differences in homing mechanisms and immune cell recruitment between early and term pregnancy, and also between the human and murine placenta. We have here mainly focused on trying to delineate how MAIT cells are recruited or retained in the intervillous space, but for future studies it would be interesting to investigate if migration of CD8^+^ EM T cells toward placental conditioned medium could be blocked by neutralizing antibodies to CXCL9, CXCL10, and CXCL11.

MIF has been described to act as a promoter of inflammation by aiding monocyte and macrophage survival by suppressing p53 (36), increasing their inflammatory response (37, 38), and by counteracting the anti-inflammatory effect of glucocorticoid hormones (39). MIF has a predominant role in the innate immune response, where for example bacterial lipopolysaccharide has been shown to induce systemic release of MIF (40) Apart from monocytes and macrophages, MIF also has a potentiating effect on the pro-inflammatory activity of T cells (26). MIF attracts monocytes by interacting with either CXCR2 or the complex CXCR2/CD74, T cells by binding CXCR4 (26) and B cells by interactions with the complex CXCR4/CD74 (41). The functional effect of the interactions between MIF and monocytes and T cells has been shown in mice with advanced atherosclerosis, where MIF-blockade led to decreased plaque infiltration by monocytes and T cells, together with a regression in plaque size (26). The mechanisms for MIF secretion is poorly understood, but it has been shown that ATP-binding cassette transporter (ABCA1) can impair secretion of MIF (42).

MIF expression has been previously observed in first and third trimester decidual cells (25, 43). NK cells isolated from first trimester decidua synthesize and secrete MIF, and MIF negatively regulates their cytolytic capacity (44). In the placenta, MIF expression has been detected in syncytiotrophoblasts, cytotrophoblasts and extravillous trophoblasts from term pregnancies (23, 25). It was recently shown that MIF has anti-apoptotic effects on trophoblasts during early pregnancy, (45) and that MIF is involved in trophoblast invasion (46). In line with our findings, the levels of MIF have been found to be higher in IVB compared to paired samples of PB in women following vaginal delivery at term pregnancy. In the same study, it was further noted that the levels of MIF in IVB was significantly lower in multigravidae women (24). Moreover, high levels of IVB MIF have been correlated to placental malaria in two cohorts (24, 47).

MAIT cells have been shown to express high levels of CXCR4 (27) and moderate levels of CCR9 (17), but low or no expression of CXCR2 (27), suggesting that CXCR4 and CCR9 are the receptors involved in the MAIT cell attracting capacity of MIF and CCL25, respectively. We have here confirmed that the majority of MAIT cells express CXCR4, and that a larger proportion of MAIT cells express CXCR4 compared to CD8^+^ EM T cells. Furthermore, the proportion of CXCR4^+^ migrating EM T cells increased when MIF was added to the migration assay, suggesting that CXCR4 is involved in the migration of T cells towards MIF. Since MAIT cells express high levels of CCR6, we evaluated blocking of the ligand CCL20, a chemokine that was also highly secreted from placental explants. However, blocking CCL20 did not significantly prevent MAIT cell migration, although there was an effect of blocking CCL20 in the majority of the experiments. This could be due to that other chemotactic factors produced by the placenta, including high levels of MIF, are more important for attracting MAIT cells in this context. On the other hand, migration of conventional EM CD8^+^ T cells was partly decreased by the addition by anti-CCL20 antibodies. The relative importance of different chemotactic factors for the migration of MAIT cells and conventional EM CD8^+^ T cells and the corresponding biological activity of chemokines is still largely unknown. It has been suggested that MAIT cells are recruited to the liver via CXCR6 and CCR6 and their ligands CXCL16 and CCL20 (48). It would be of great interest to study and compare MAIT cell and EM T cell migration in different physiological settings, including the intervillous space and the liver sinusoids, which are both characterized by low pressure blood flow.

Levels of CCL21 and CCL27 were significantly higher in PB compared to IVB, and inversely related to MAIT cell proportions in IVB. CCL21 is the ligand to CCR7, a chemokine receptor that is highly expressed by naïve T cells and is important for homing to lymph nodes. This is in accordance with the higher proportion of naïve T cells in PB compared to IVB, which are constantly circulating in PB to visit lymph nodes. CCL27 is predominantly expressed by keratinocytes and has been shown to attract CCR10^+^ T cells to the skin (49), and MAIT cells only express low levels of CCR10 (50). For future studies, it would be interesting to further study the reverse relationship between low MAIT cell proportions and high levels of CCL21 and CCL27 in IVB.

One limitation with this study is that the number of migrating MAIT cells and CD8^+^ EM T cells in the migration assay sometimes were low, which can question the relevance of the results. Indeed, in the assays in which the chemoattractive capacity of MIF was investigated, many of the experiments contained low numbers of migrating MAIT cells. The majority of the experiments were performed with purified T cells, but we also included six experiments where CD4-depleted T cells were added to the transwell. In this way, the cells that were put in the migration assay were enriched for MAIT cells and CD8^+^ EM T cells, which consequently also led to higher migrating cell counts. Although MIF appeared to have an attracting effect on T cells in general, as determined by cell counts, the proportion of MAIT cells among migrating CD3^+^ was increased after addition of MIF. In contrast, proportions of CD8^+^ EM T cells were unaltered in the presence of MIF. Thus, although the robustness of the flow cytometry-based migration assay might be questioned, the results are still in line with the positive correlation between levels of MIF and proportions of MAIT cells in IVB.

The physiological role of MAIT cells in the placenta and their importance during pregnancy is not known. One possible function is protection of the fetus against bacterial and fungal infections, but also against viral infections since MAIT cells are also activated by inflammatory signals in an MR1-independent manner (11, 12, 14). Their rapid cytotoxic effects could provide an efficient innate-like defense mechanism to prevent pathogens from crossing the fetal-maternal barrier. The direct anti-bacterial and tissue protective activity of MAIT cells mediated by IL-17 and IL-22 production, (19), respectively, may also be important. Since many commensal and pathogenic microbes synthesize riboflavin, whereas human cells do not, MAIT cells provide an essentially different mechanism of discrimination between self and non-self compared to conventional T cells (5).

We have previously found that MAIT cells in IVB respond more vigorously with production of cytokines and cytotoxic molecules compared to peripheral blood MAIT cells after stimulation with *Escherichia coli*, (4) further indicating their importance in mediating anti-microbial inflammatory responses in placental tissues. On the other hand, it can be speculated that aggravated MAIT cell activation in intervillous space could be involved in initiation of preterm birth or play a role in other pregnancy complications. It was recently shown that the proportion of MAIT cells in peripheral blood of patients with pre-eclampsia was lower compared to non-complicated pregnancies (51), indicating that MAIT cells may have accumulated in the placenta and that they possibly could be involved in mediating the pathophysiological traits of pre-eclampsia. It is therefore interesting that patients who later developed pre-eclampsia had low levels of MIF in PB at gestational week 13 (52), which was followed by a subsequent pathological rise in MIF in patients with pre-eclampsia, and the highest levels were seen in the patients who developed pre-eclampsia before gestational week 34 (53). This warrants further studies of the role of MIF, MAIT cell homing, and MAIT cell function at the fetal maternal interface in the context of pregnancy complications.

Our data suggests that certain immune cell subsets are enriched in the intervillous space in healthy term placentas. These findings challenge the general conception about placental blood circulation, since the entire blood content does not seem to be exchanged. Since the intervillous blood is surrounded by the fetal placenta, this compartment may be optimal for studying immune dysregulation and for identifying immunological factors involved in complicated pregnancies.

## Ethics Statement

This study was carried out in accordance with the recommendations of the regional review board of ethics in research at Karolinska Institutet with written informed consent from all subjects. All subjects gave written informed consent in accordance with the Declaration of Helsinki. The protocol was approved by the regional review board of ethics in research at Karolinska Institutet.

## Author Contributions

MS conceived, designed, performed, and analyzed the experiments, interpreted the results and wrote the paper. LG performed and analyzed the experiments and interpreted the results. ET and SG interpreted the results. EL and JD designed and performed the experiments. JS designed the experiments and interpreted the results. IM designed the experiments and interpreted the results. A-CL performed all multivariate factor analyses and interpreted the results. HK conceived, designed and analyzed the experiments, interpreted the results, wrote the paper and was the principal investigator of the project. All authors participated in final approval of the manuscript.

### Conflict of Interest Statement

The authors declare that the research was conducted in the absence of any commercial or financial relationships that could be construed as a potential conflict of interest.
