# Supplementary material for: Recruitment of MAIT Cells to the Intervillous Space of the Placenta by Placenta-Derived Chemokines
Source: Front Immunol. 2019 Jun 6;10:1300. doi: 10.3389/fimmu.2019.01300 (PMC6563723; doi:10.3389/fimmu.2019.01300)
Supplement: Supplementary file 1 [file Data_Sheet_1.docx]

**Supplementary figure S1
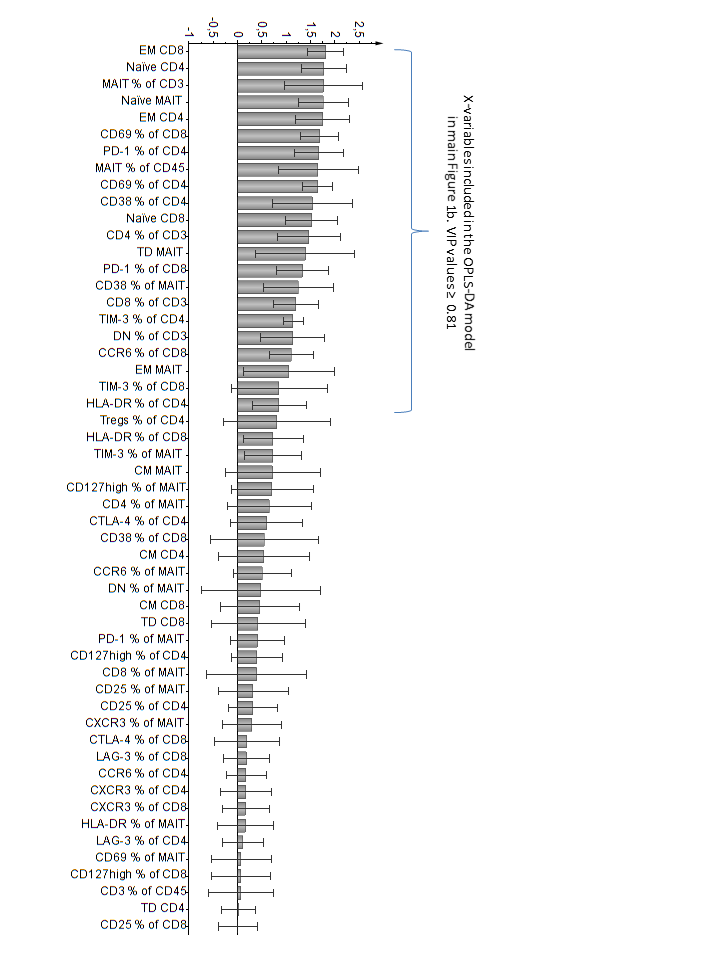
**

**Supplementary Figure S1**. Variable influence on projection (VIP) plot. The VIP plot displays the VIP values as a column plot sorted in descending order with confidence intervals derived from jack knifing. VIP summarizing the importance of the X-variables that contribute most (positively or negatively) to a separation between peripheral and intervillous blood based on T cell phenotypes (PB, *n* = 25 and IVB, *n* = 24). Blue bar indicates factors with a VIP-values >0.81, which are presented in main Fig 1b.

**Supplementary figure S2**

**
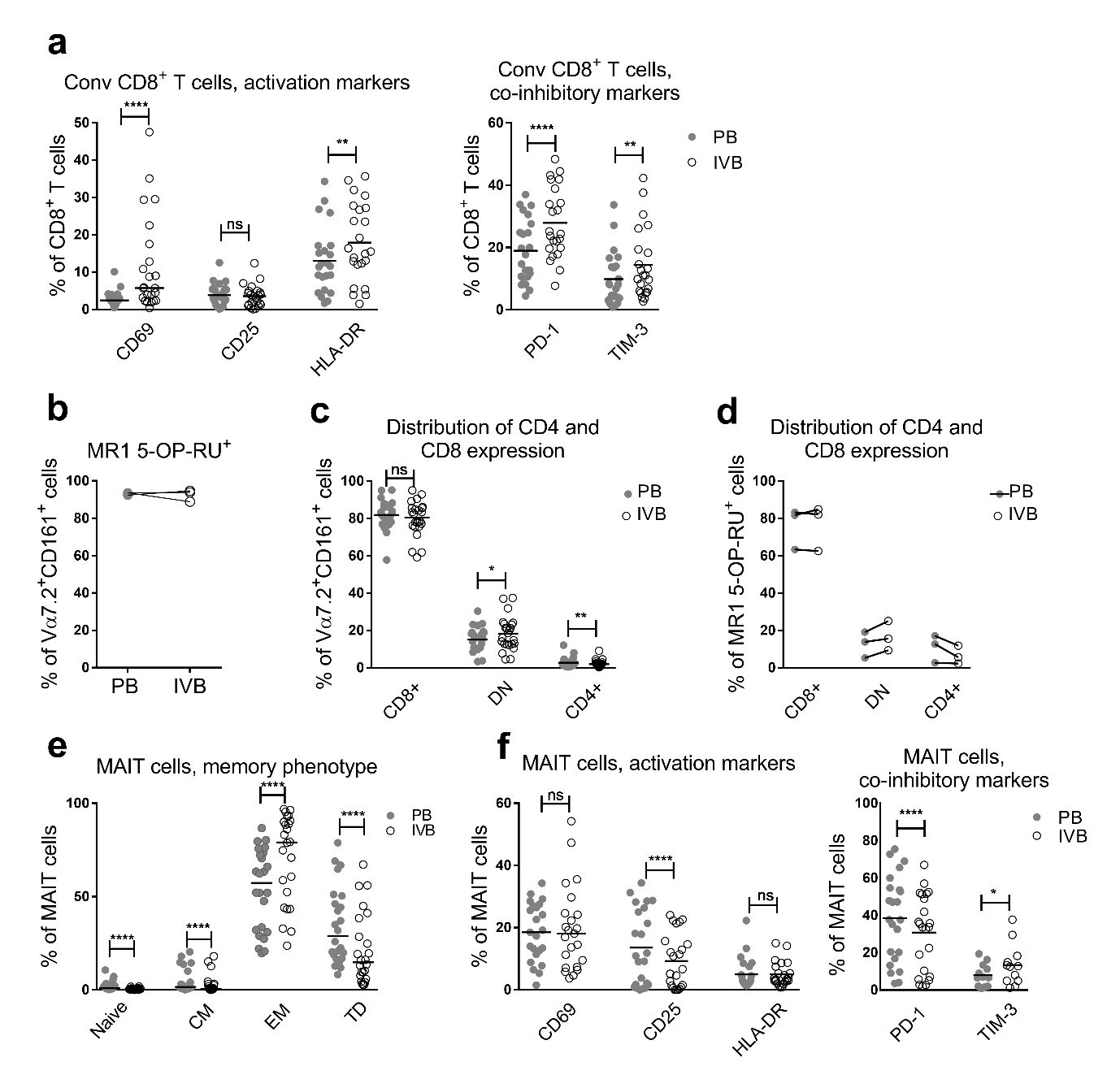
Supplementary Figure S2. CD8^+^ T cell and MAIT cell phenotype in intervillous and peripheral blood.** (a) The expression of CD69, CD25 and HLA-DR (left) and PD-1 and TIM-3 (right) on conventional (conv) CD8^+^ T cells compared between paired samples of intervillous (IVB) and peripheral blood (PB) (*n* = 23). (b) Proportion of Vα7.2^+^CD161^+^ cells binding to the MR1 5-OP-RU tetramer (*n* = 3). (c) Vα7.2^+^CD161^+^ MAIT cell subsets based on the surface expression of CD8^+^, CD4^-^CD8^-^ (DN) or CD4^+^ compared between paired samples of IVB and PB (*n* = 24). Distribution of CD8^+^, DN and CD4^+^ cells in MR1 5-OP-RU tetramer positive cells (*n* = 3). (d) MAIT cell proportions of naïve, central memory (CM), effector memory (EM) or terminally differentiated (TD) based on the expression of CCR7 and CD45RA, in paired samples of IVB and PB (*n* = 24). (f) The expression of CD69, CD25 and HLA-DR (*n* = 23) (left), and PD-1 (*n* = 23) and TIM-3 (*n* =12) (right) on MAIT cells compared between paired samples of IVB and PB. Line in graphs represents the median. Comparisons between paired samples were made using the nonparametric Wilcoxon test. ns = not significant; * *p* < 0.05; ** *p* < 0.01; **** *p* < 0.0001.

**Supplementary figure S3**

**
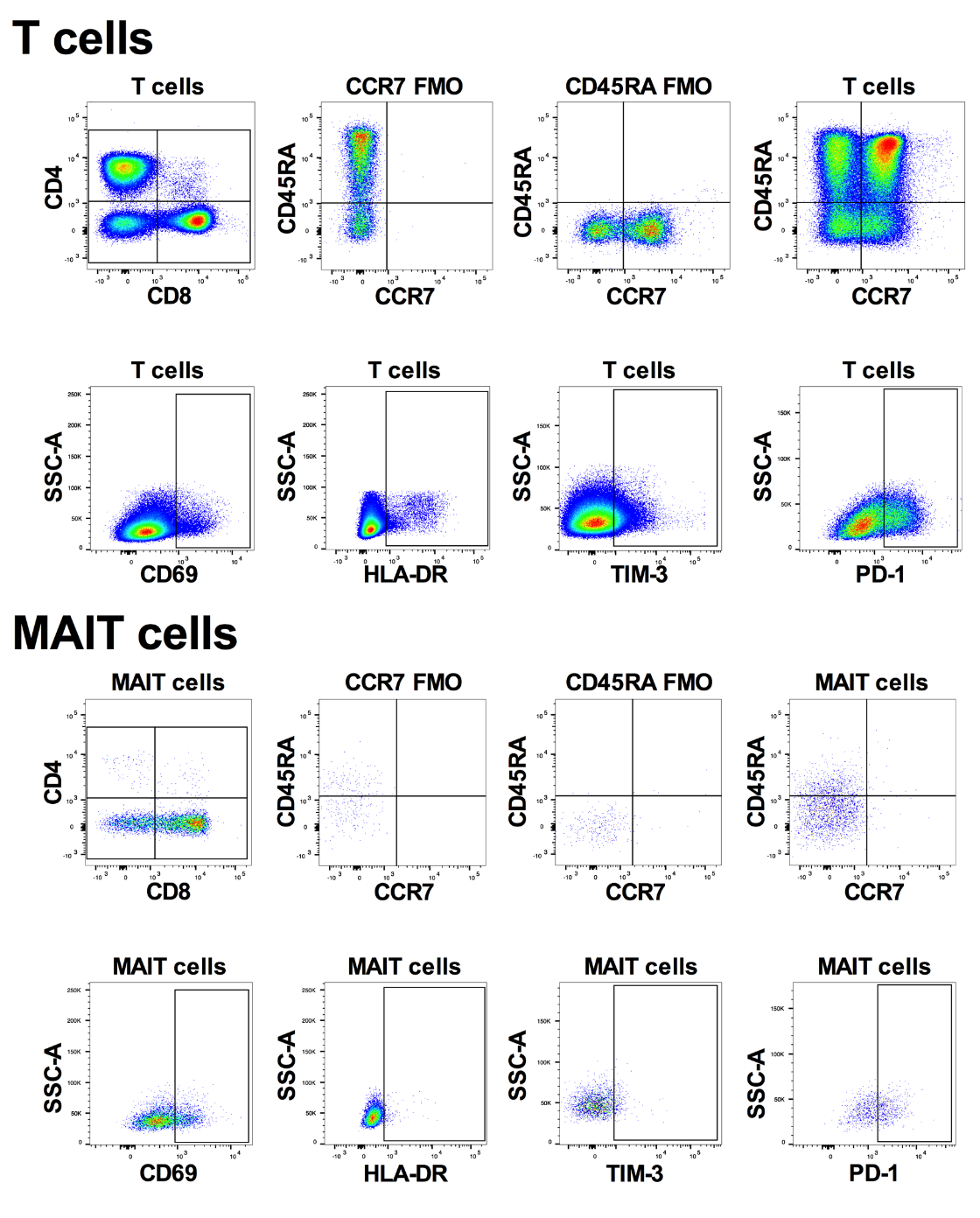
**

**Supplementary Figure S3.** Representative FACS plots showing gating strategies for the indicated markers on conventional T cells and MAIT cells.

**Supplementary figure S4**

**
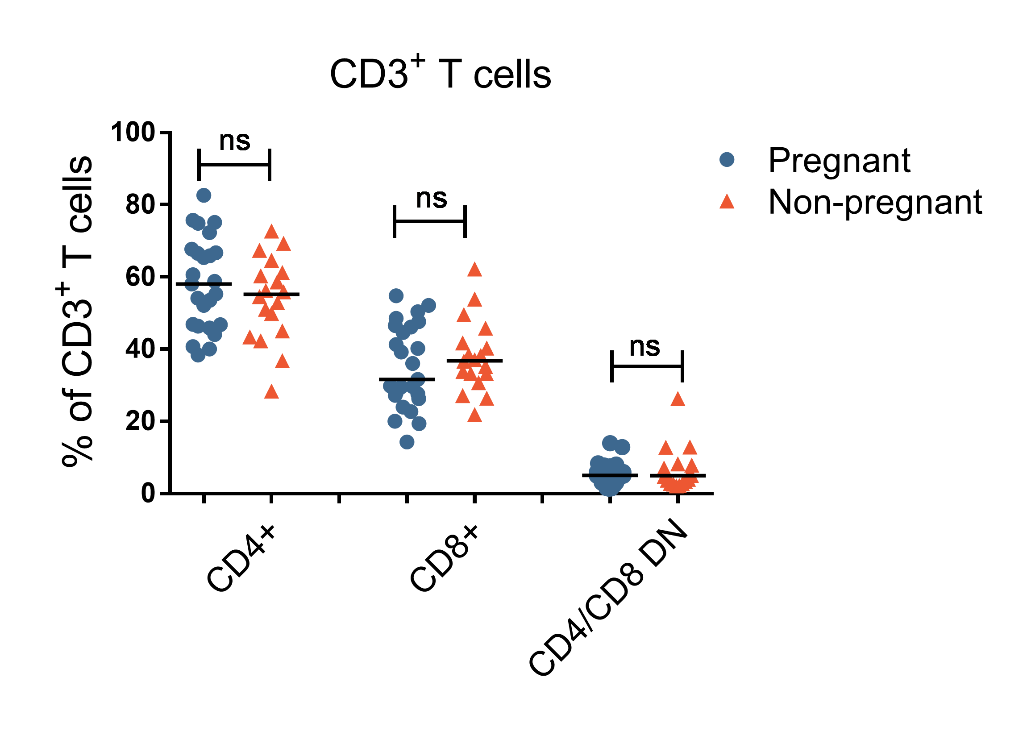
**

**Supplementary Figure S4. T cell composition in peripheral blood of pregnant and non-pregnant women.** Percentage of CD4+, CD8+ and double-negative (DN) T cells out of total CD3^+^ cells compared between peripheral blood from pregnant (*n* = 35) and non-pregnant women (*n* = 18). Comparisons were performed by Mann-Whitney test. ns = not significant

**Supplementary figure S5**

**
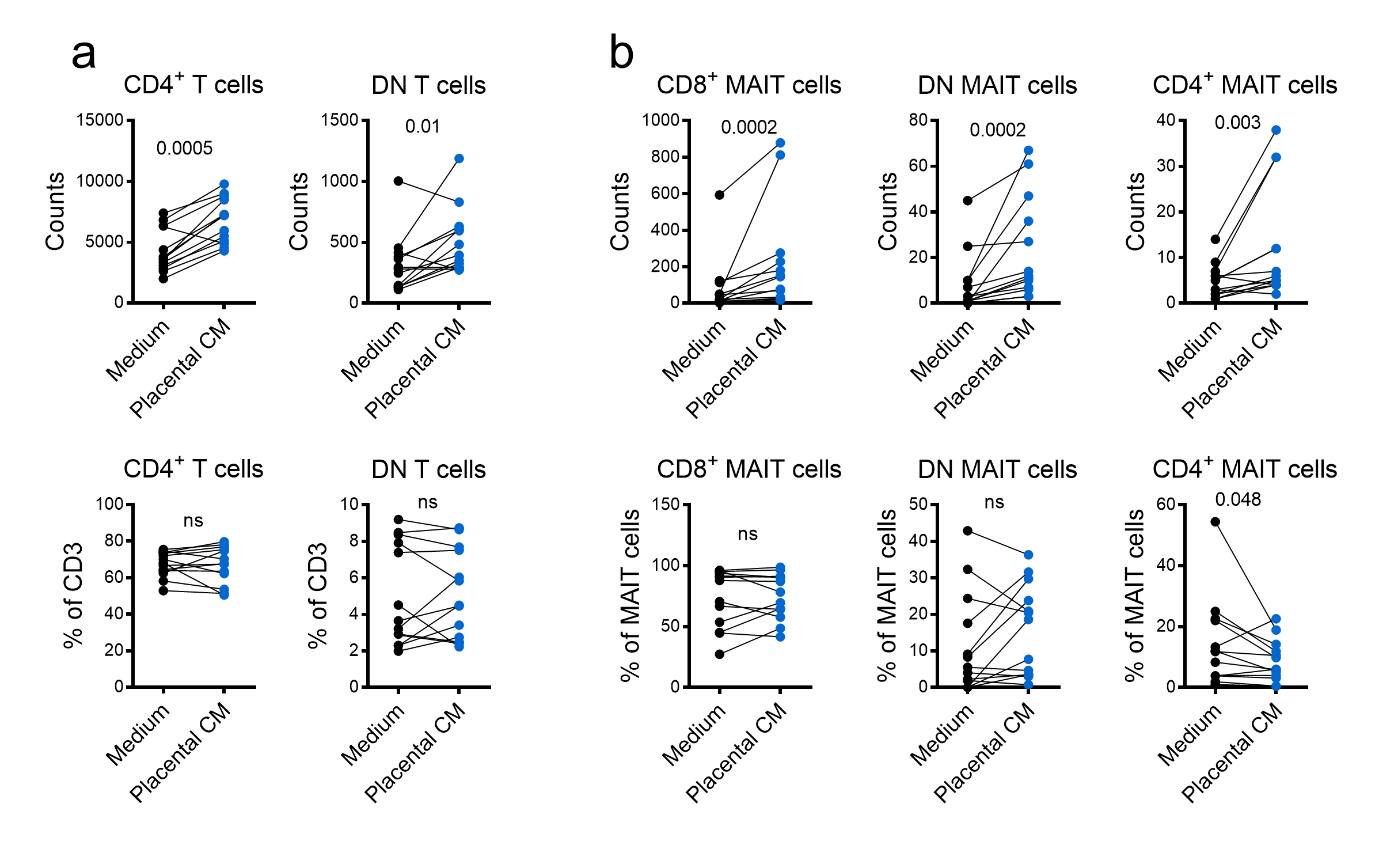
**

**Supplementary Figure S5. Migration of T cell and MAIT cell subsets towards placental conditioned medium.** Migration of a) conventional CD4^+^ and CD4 and CD8 double-negative (DN) T cells and b) CD8^+^, DN and CD4^+^ MAIT cells towards placental conditioned medium (CM). The top row shows the number of migrated cells and the bottom depicts proportions of indicated T cell or MAIT cell subsets within CD3^+^ T cells or MAIT cells, respectively. Paired experiments are connected by a line. Comparisons between paired samples were made using the nonparametric Wilcoxon test. ns = not significant.

**Supplementary figure S6**

**
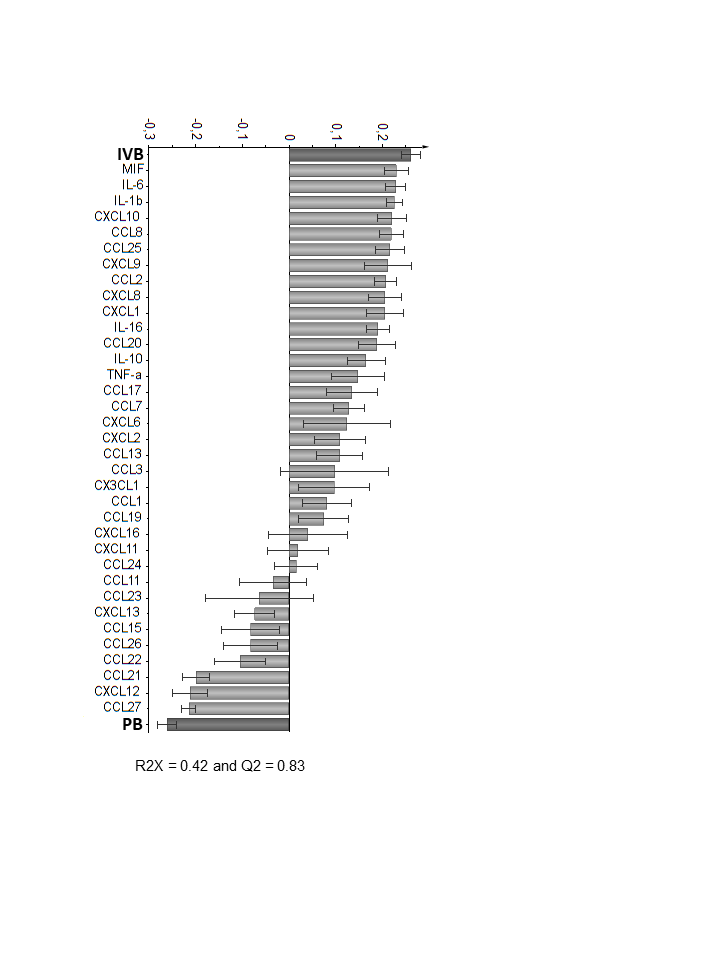
**

**Supplementary Figure S6. Chemokine profile in intervillous blood is distinct from that of peripheral blood.** OPLS-DA loading plot displaying a separation between peripheral (PB) and intervillous blood (IVB) based on the measured levels of 35 chemokines and cytokines (*n* = 25).

**Supplementary figure S7**

**
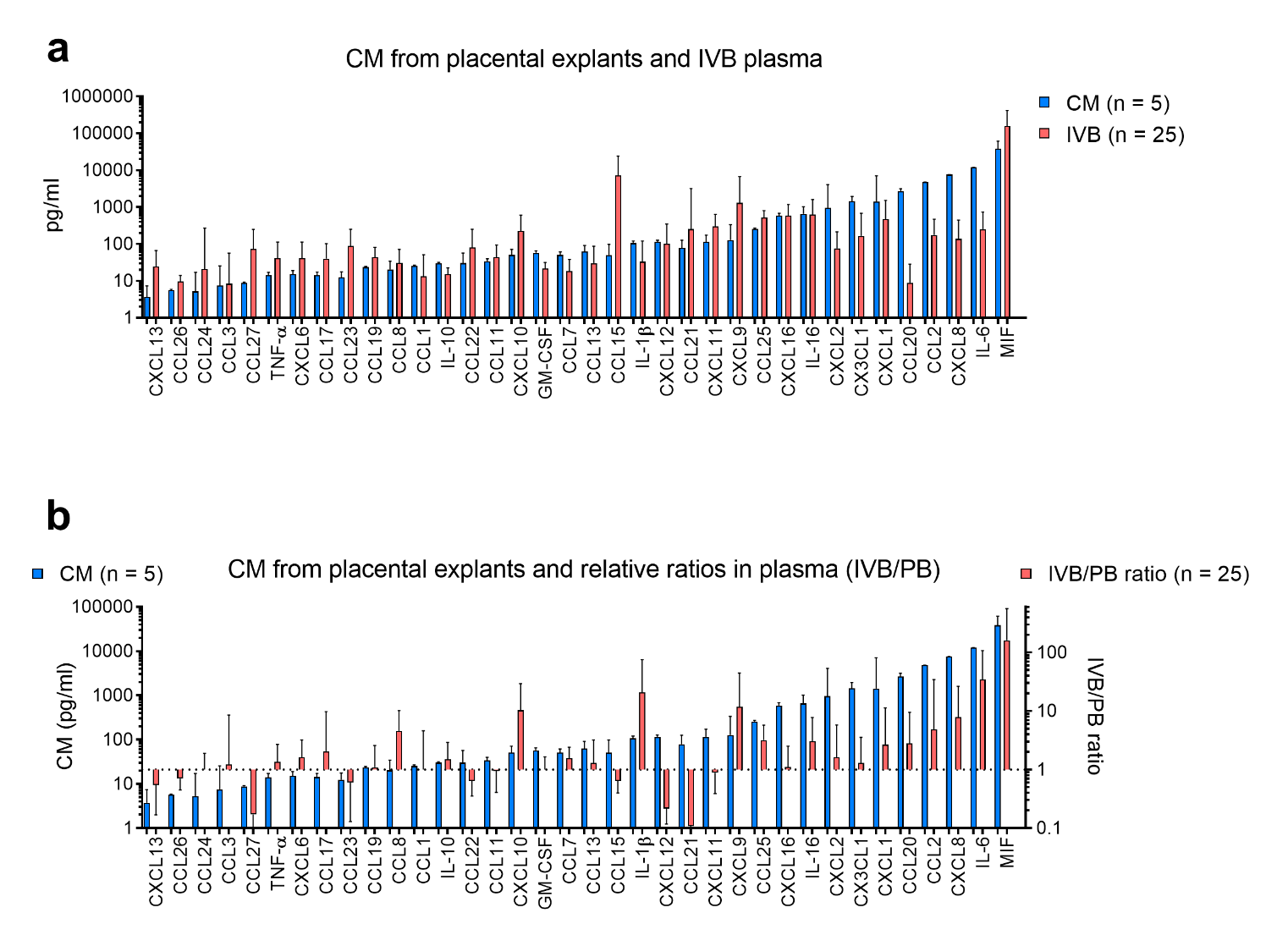
**

**Supplementary Figure S7. Chemokines and cytokines in conditioned medium (CM) from placental explants and intervillous blood (IVB) plasma.** a) Levels of chemokines and cytokines in the placental CM and IVB plasma sorted on lowest to highest levels in CM. b) The relative ratio between levels of chemokines and cytokines in IVB divided by levels in peripheral blood (PB) plasma (IVB/PB ratio) is shown on the right y-axis, side by side with the data on placental CM on the left y-axis. CM data is same as shown in Fig 2a, and IVB and PB plasma levels are based on the data shown in Fig 3a. Bars depict the median value, and the error bars the upper range. For 3 out of the 5 CM donors, data from IVB was available from the same donor.

**Supplementary figure S8**

**
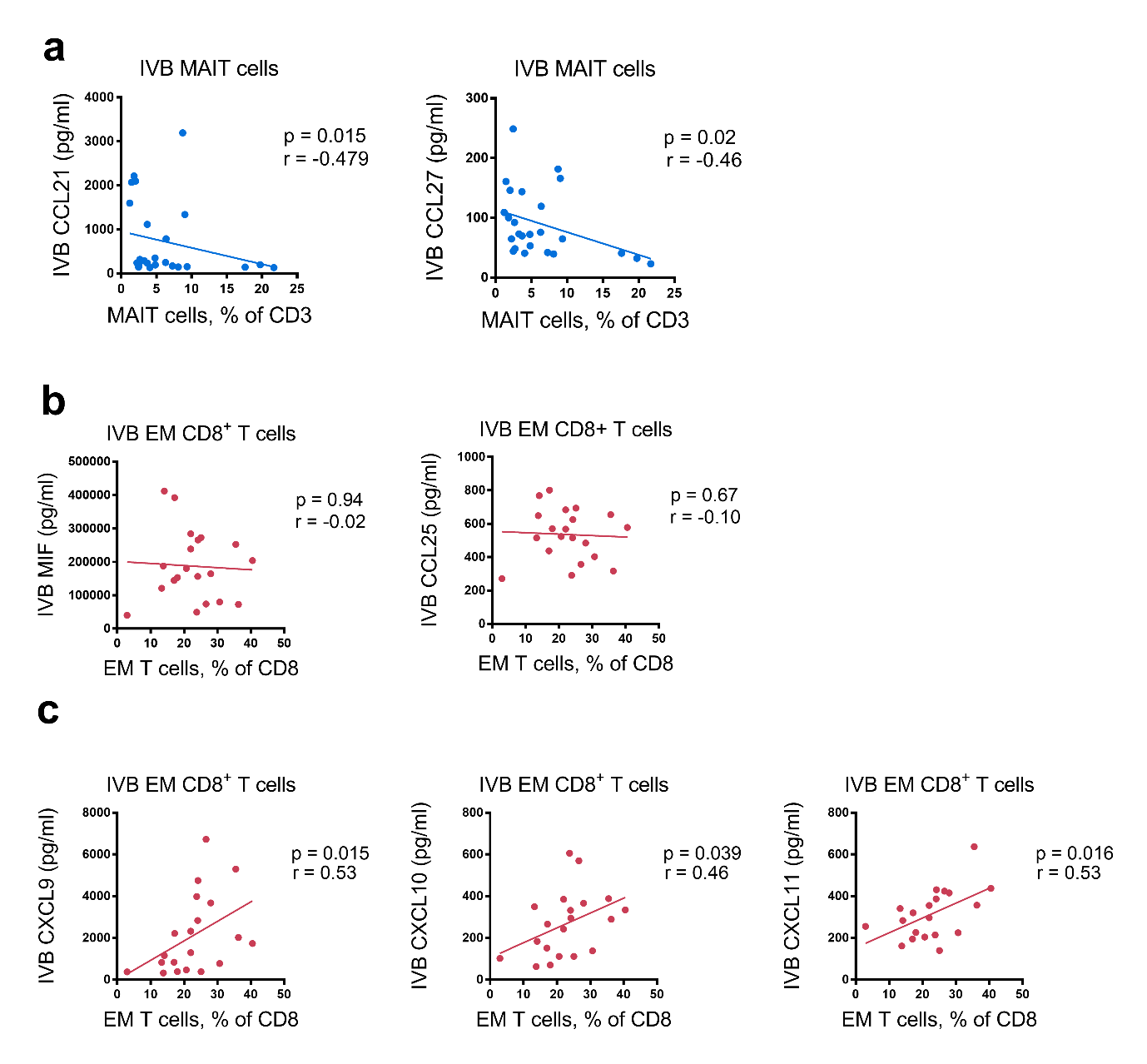
**

**Supplementary Figure S8. The frequency of CD8^+^ effector memory T cells from intervillous blood correlates to levels of CXCR3 chemokines in intervillous plasma.** (a) Intervillous blood (IVB) MAIT cells were inversely correlated to IVB levels of CCL21 and CCL27 (*n* = 25) (b) The frequency of CD8^+^ effector memory (EM) T cells from IVB does not correlate to either MIF (left) or CCL25 (right) levels in IVB plasma (*n* = 20). (b) The frequency of CD8^+^ EM T cells from IVB correlates to levels of CXCL9 (left), CXCL10 (middle), and CXCL11 (right) in IVB plasma (*n* = 20). Correlations between paired samples were made using the nonparametric Spearman correlation test.

**Supplementary figure S9**

**
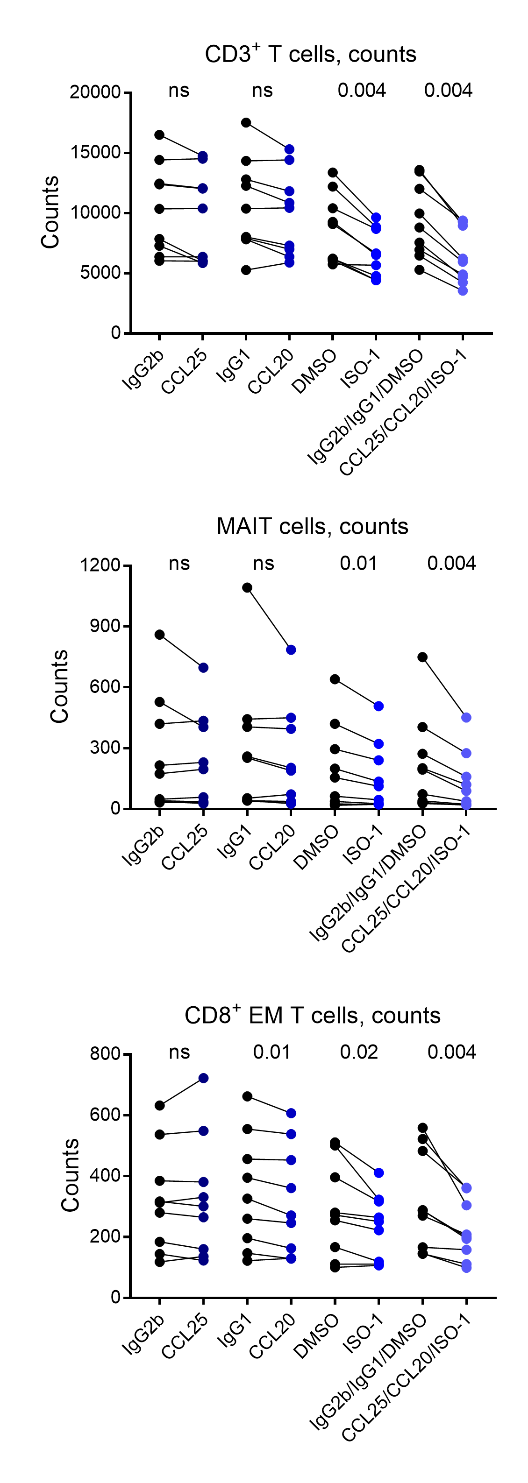
**

**Supplementary Figure S9. Migration of T cell subsets towards placental conditioned medium can be partially inhibited by neutralization of chemokines.** The effect of blocking CCL25, CCL20 and MIF, alone or all together, on the migration of CD3^+^ T cells (top), MAIT cells (middle) or CD8^+^ effector memory (EM) T cells (bottom), towards placental conditioned medium. Results are expressed as cell counts after addition of isotype or diluent control (DMSO) or neutralizing reagents. Paired samples are shown by connecting lines. Comparisons between paired samples were made using the nonparametric Wilcoxon test. ns = not significant.

**Supplementary figure S10**


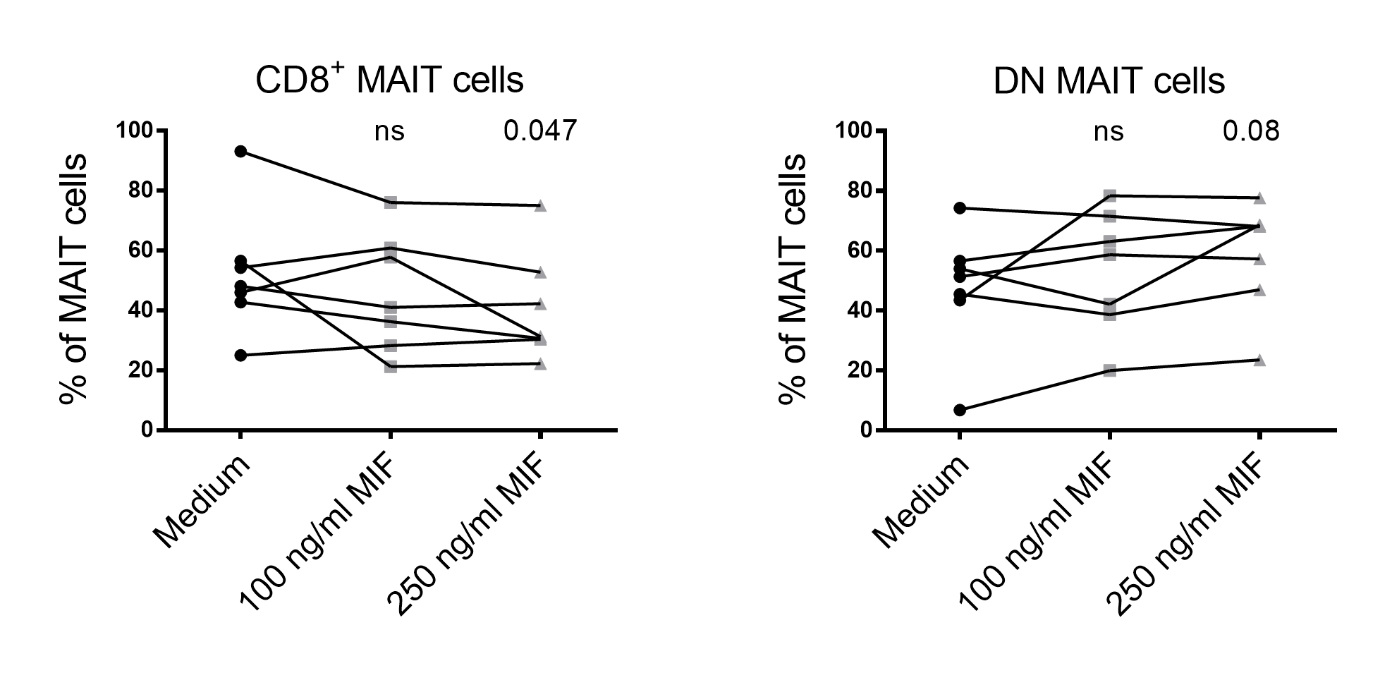


**Supplementary Figure S10. Migration of MAIT cell subsets towards MIF.** Proportions of migrating CD8^+^ (left) and DN (right) MAIT cells in the absence or presence of increasing concentrations of recombinant MIF. Paired samples are shown by connecting lines. Comparisons between paired samples were made using the nonparametric Wilcoxon test. ns = not significant.

**Supplementary Table S1. Antibodies and viability dye used for flow cytometry.**

| **Surface markers** | **Fluorochrome** | **Clone** | **Source** |
| --- | --- | --- | --- |
| CD3 | V450 | UCHT1 | BD |
| CD3 | FITC | UCHT1 | BD |
| CD3 | PE-Cy7 | UCHT1 | BD |
| CD4 | V500 | RPA-T4 | BD |
| CD4 | A700 | RPA-T4 | BD |
| CD8 | A700 | RPA-T8 | BD |
| CD8 | APC | SK1 | BD |
| CD8 | PE-Cy7 | RPA-T8 | BD |
| CD8 | APC-Cy7 | SK1 | BD |
| CD25 | Bv421 | M-A251 | BD |
| CD25 | PE | M-A251 | BD |
| CD38 | Bv421 | HIT2 | BD |
| CD45 | APC-H7 | 2D1 | BD |
| CD45RA | PE-Cy7 | HI100 | BD |
| CD69 | FITC | L78 | BD |
| CD69 | APC | L78 | BD |
| CD127 | APC-A700 | R34.34 | Beckman Coulter |
| CD152 (CTLA-4) | PE | BNI3 | BD |
| CD152 (CTLA-4) | FITC | A3.4H2.H12 | LSBio |
| CD161 | PE | HP-3G10 | BioLegend |
| CD183 (CXCR3) | APC | 1C6/CXCR3 | BD |
| CD184 (CXCR4) | Bv421 | 12G5 | BioLegend |
| CD196 (CCR6) | FITC | G034E3 | BioLegend |
| CD197 (CCR7) | PE-CF594 | 150503 | BD |
| CD223 (LAG-3) | FITC | 17B4 | LSBio |
| CD223 (LAG-3) | PE | REA351 | Miltenyi |
| CD279 (PD-1) | BV421 | EH12.1 | BD |
| CD366 (TIM-3) | APC | F38-2E2 | Miltenyi |
| HLA-DR | FITC | G46-6 | BD |
| TCR Vα7.2 | APC-Cy7 | 3C10 | BioLegend |
| - | 7AAD | - | BD |

Abbreviations; CD, Cluster of differentiation, BD, BD Biosciences (Franklin Lakes, NJ), FITC, Fluoresceinisothiocyanate, Pe-Cy7, Phycoerythrin-cyanine 7, A700, Alexa Fluor 700, APC, Allophycocyanine, APC-Cy7, Allophycocyanine-indo tricarbocyanine, BV421, Brilliant violet 421, PE, Phycoerythrin, APC-H7, Allophycocyanine H7, APC-A700, Allophycocyanine Alexa Fluor 700, Beckman Coulter, Beckman Coulter (Fullerton, CA), LSBio, LSBio (Seattle WA), BioLegend, BioLegend (San Diego, CA), PE-CF594, Phycoerythrin-CF 594, Miltenyi, Miltenyi Biotec (Bergisch Gladbach, Germany).

**Supplementary Table S2. Luminex assay performance**

| **Chemokine/cytokine** | **Sensitivity***  **(limit of detection)** | **Lower limit**  **of quantification*** | **Upper limit**  **of quantification*** |
| --- | --- | --- | --- |
| MIF | 15.4 | 23.1 | 377 724 |
| IL-6 | 0.1 | 0.7 | 12 000 |
| IL-1β | 0.1 | 0.4 | 7 000 |
| CXCL10 | 1.1 | 1.6 | 7 714 |
| CCL8 | 0.04 | 0.3 | 4 056 |
| CCL25 | 4.9 | 20.6 | 114 493 |
| CXCL9 | 1.1 | 1.8 | 19 600 |
| CCL2 | 0.1 | 0.3 | 4 812 |
| CXCL8 | 0.04 | 0.5 | 7 640 |
| CXCL1 | 4.2 | 3.1 | 7 024 |
| IL-16 | 0.8 | 2.1 | 34 000 |
| CCL20 | 0.1 | 0.3 | 4 675 |
| IL-10 | 0.9 | 1.3 | 18 708 |
| TNF-α | 0.2 | 0.9 | 13 879 |
| CCL17 | 1.1 | 1.7 | 430 |
| CCL7 | 1.3 | 1.9 | 20 133 |
| CXCL6 | 0.6 | 0.8 | 11 135 |
| CXCL2 | 2.7 | 4.6 | 13 257 |
| CCL13 | 0.1 | 0.2 | 3 368 |
| CCL3 | 0.3 | 0.4 | 1 543 |
| CX3CL1 | 0.9 | 4.0 | 11 463 |
| CCL1 | 1.6 | 1.8 | 1 015 |
| CCL19 | 1.1 | 3.0 | 48 494 |
| CXCL16 | 0.1 | 0.5 | 2 867 |
| CXCL11 | 0.05 | 0.1 | 2 298 |
| CCL24 | 3.2 | 6.2 | 4 073 |
| CCL11 | 0.7 | 1.5 | 3 859 |
| CCL23 | 0.5 | 1.0 | 14 450 |
| CXCL13 | 0.1 | 0.7 | 1 200 |
| CCL15 | 0.2 | 1.7 | 9 100 |
| CCL26 | 0.5 | 0.9 | 12 109 |
| CCL22 | 0.5 | 0.9 | 14 649 |
| CCL21 | 12 | 21.9 | 3 923 |
| CXCL12 | 10.3 | 8.3 | 115 730 |
| CCL27 | 0.3 | 1.2 | 5 000 |

*pg/ml
